# Change in thermal transitions and water uptakes of poly(l-lactic acid) blends upon hydrolytic degradation

**DOI:** 10.1016/j.dib.2016.11.088

**Published:** 2016-11-28

**Authors:** Hideko T. Oyama, Daisuke Tanishima, Shintaro Maekawa

**Affiliations:** a Department of Chemistry, College of Science, Rikkyo University, 3-34-1 Nishi-Ikebukuro, Toshima-ku, Tokyo 171-8501, Japan; b School of Chemical Engineering, Fuzhou University, No. 2 Xueyuan Rd, Minhou County, Fuzhou 350116, People׳s Republic of China; c R&D Center, Mitsui Chemicals Inc., 580-32 Nagaura, Sodegaura, Chiba 299-0265, Japan

## Abstract

This article reports experimental data related to the research article entitled “Poly(malic acid-co-l-lactide) as a Superb Degradation Accelerator for Poly(l-lactic acid) at Physiological Conditions” (H.T. Oyama, D. Tanishima, S. Maekawa, 2016) [1]. Hydrolytic degradation of poly(l-lactic acid) (PLLA) blends with poly(aspartic acid-co-l-lactide) (PAL) and poly(malic acid-co-l-lactide) (PML) oligomers was investigated in a phosphate buffer solution at 40 °C. It was found in the differential scanning calorimetry measurements that upon hydrolysis the cold crystallization temperature (*T_c_*) and the melting temperature (*T_m_*) significantly shifted to lower temperature. Furthermore, the hydrolysis significantly promoted water sorption in both blends.

**Specifications Table**

**Table:** 

Subject area	*Chemistry*
More specific subject area	*Polymer*
Type of data	*figure*
How data was acquired	A differential scanning calorimeter, TA Instruments DSC-Q200 (manufactured in USA), was employed at a heating rate of 10 °C/min. Water uptakes of the blend films during hydrolysis of PLLA blends were estimated from Eq. (1), where the mass of hydrolyzed specimens, followed by the removal of the buffer solution without and with drying in vacuo are *m*_*w*_ and *m*_*d*_, respectively;
	(1) $$\;\mathrm{Water}\;\mathrm{uptake}\;\left(wt\%\right)=\left(m_{w}–m_{d}\right)/m_{d}x\;100$$
Data format	Raw
Experimental factors	None
Experimental features	Hydrolytic degradation of poly(l-lactic acid) (PLLA) blends with poly(aspartic acid-co-l-lactide) (PAL) [2] and poly(malic acid-co-l-lactide) (PML) [3] oligomers was carried out, immersing the blend films in a phosphate buffer solution (pH 7.4) at 40 °C. Change in thermal transitions and water uptakes of PLLA blends upon hydrolytic degradation was investigated.
Data source location	Tokyo, Japan
Data accessibility	The data is with this article.

**Value of the data**

1. In the past, the advanced stages in hydrolysis of PLLA were studied at elevated temperature or alkaline conditions, since the reaction proceeds very slowly at physiological conditions. But the present study was carried out at physiological conditions in the presence of oligomeric degradation accelerators so that the results obtained here are directly useful for biopharmaceuticals and tissue regeneration.
2. There are no studies on PLLA blends with PML in the literature except for Ref. [1].
3. There are few reports on how thermal transitions (e.g., *T_g_*, *T_c_*, and *T_m_*) of PLLA blends are changed upon hydrolysis such as Fig. 1.
4. Hydrolysis rate constant of PLLA at 20 wt% loading is enhanced 15 times by PAL and 34 times by PML,^1^ which reaction is initiated by water sorption. So it is essential to measure water uptakes at different hydrolysis stages, like Fig. 2.

## 1 Data

**Fig. 1:**
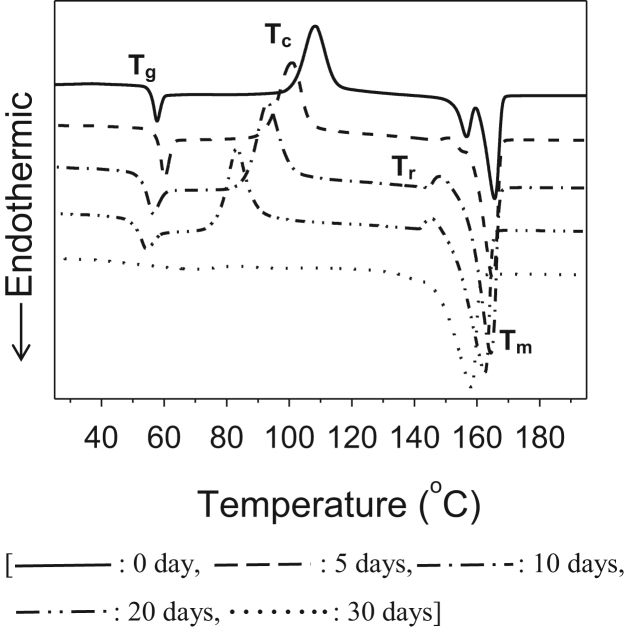
Differential scanning calorimetry (DSC) thermograms of (90/10) PLLA/PML after hydrolysis for different times, where *T_g_*, *T_c_*, *T_r_*, and *T_m_* are the glass transition temperature, the cold crystallization temperature, the recrystallization temperature, and the melting temperature, respectively.

**Fig. 2:**
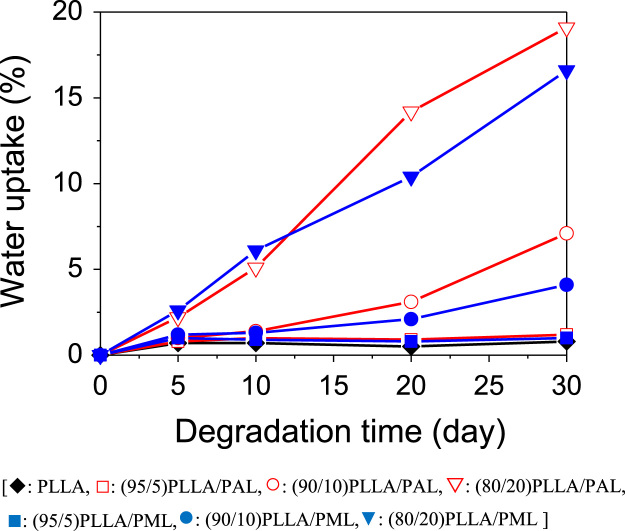
Change in water uptake of neat PLLA and PLLA blends during immersion in a phosphate buffer solution (pH=7.4, 40 °C).

Hydrolytic degradation of PLLA blends with biological safe oligomers, PML and PAL, at physiological conditions was investigated in the present study. Change in differential scanning calorimetry (DSC) thermograms (Fig. 1) and the water uptakes (Fig. 2) upon hydrolytic degradation was shown.

## 2 Experimental design, materials and methods

Poly(l-lactic acid) (PLLA) (*M*_*n*_=1.3×10^5^, *M*_*w*_=2.2×10^5^, [d-lactyl unit]=1.4%) was melt-blended with 5, 10, and 20 wt% of either PAL or PML (molar ratio of l- lactyl to aspartic acid or malic acid units=10, their *M*_*n*_*=* 1.6×10^3^ and *M*_*w*_=3.5×10^3^) at 175 °C (PLLA/PML) or 185 °C (PLLA/PAL) for 5 min with a rotation speed of 50 rpm using a twin blade mixer (Toyo Seiki, Labo Plastomill 4M150 equipped with KF70V2, manufactured in Japan). The blend films with *ca*. 500 μm thickness were prepared by compression molding and used for hydrolytic degradation tests in a phosphate buffer solution at 40 °C. Changes in DSC thermograms and water uptakes were monitored during the hydrolysis of the PLLA blends.

## References

[1] H.T. Oyama, D. Tanishima, S. Maekawa, Poly(malic acid-co-L-lactide) as a superb degradation accelerator for poly(L-lactic acid) at physiological conditions, *Polym. Degrad. Stabil.* 134 (2016) 265-271.

[2] Shinoda H., Asou Y., Suetsugu A., Tanaka K. (2003). Synthesis and characterization of amphiphilic biodegradable copolymer, poly(aspartic acid-co-lactic acid). Macromol. Biosci..

[3] S. Maekawa, H. Onishi, S. Usugi, (Mitsui Chemicals Co. Ltd), Biodegradable resin composition and molded article of the same, JP WO2012/13768110.11), 2012.

